# Increasing comorbidity is associated with worsening physical function and pain after primary total knee arthroplasty

**DOI:** 10.1186/s12891-016-1261-y

**Published:** 2016-10-07

**Authors:** Maren E. Hilton, Terence Gioe, Siamak Noorbaloochi, Jasvinder A. Singh

**Affiliations:** 1 Rheumatology Section, Medicine Service and Division of Rheumatology, Department of Medicine, University of Minnesota, Minneapolis, MN USA; 2 Department of Orthopedic Surgery, VA Medical Center, Minneapolis, MN USA; 3 Division of General Internal Medicine, VA Medical Center, Minneapolis, MN USA; 4 Medicine Service, VA Medical Center, Birmingham, AL USA; 5 Department of Medicine at the School of Medicine, and Division of Epidemiology at the School of Public Health, University of Alabama at Birmingham, Faculty Office Tower 805B, 510 20th Street S, Birmingham, AL 35294 USA; 6 Department of Orthopedic Surgery, Mayo Clinic College of Medicine, Rochester, MN USA; 7 Present address: Arthritis and Rheumatology Consultants, 7250 France Ave #215, Edina, 55435 MN USA

**Keywords:** Comorbidity, Physical Function, Pain, Primary Total Knee Arthroplasty, TKA, Worsening

## Abstract

**Background:**

Previous studies suggested that pre-operative comorbidity was a risk factor for worse outcomes after TKA. To our knowledge, studies have not examined whether postoperative changes in comorbidity impact pain and function outcomes longitudinally. Our objective was to examine if increasing comorbidity postoperatively is associated with worsening physical function and pain after primary total knee arthroplasty (TKA).

**Methods:**

We performed a retrospective chart review of veterans who had completed Western Ontario and McMaster Universities Osteoarthritis Index (WOMAC) and Short Form-36 (SF36) surveys at regular intervals after primary TKA. Comorbidity was assessed using a variety of scales: validated Charlson comorbidity index score, and a novel Arthroplasty Comorbidity Severity Index score (Including medical index, local musculoskeletal index [including lower extremity and spine] and TKA-related index subscales; higher scores are worse ), at multiple time-points post-TKA. We used mixed model linear regression to examine the association of worsening comorbidity post-TKA with change in WOMAC and SF-36 scores in the subsequent follow-up periods, controlling for age, length of follow-up, and repeated observations.

**Results:**

The study cohort consisted of 124 patients with a mean age of 71.7 years (range 58.6–89.2, standard deviation (SD) 6.9) followed for a mean of 4.9 years post-operatively (range 1.3–11.4; SD 2.8). We found that post-operative worsening of the Charlson Index score was significantly associated with worsening SF-36 Physical Function (PF) (beta coefficient (ß) = -0.07; *p* < 0.0001), SF-36 Bodily Pain (BP) (ß = -0.06; p = 0.002), and WOMAC PF subscale (ß = 0.08; *p* < 0.001; higher scores are worse) scores, in the subsequent periods. Worsening novel medical index subscale scores were significantly associated with worsening SF-36 PF scores (ß = -0.03; *p* = 0.002), SF-36 BP (ß = -0.04; *p* < 0.001) and showed a non-significant trend for worse WOMAC PF scores (ß = 0.02; *p* = 0.11) subsequently. Local musculoskeletal index subscale scores were significantly associated with worsening SF-36 PF (ß = -0.05; *p* = 0.001), SF-36 BP (ß = -0.04; *p* = 0.03) and WOMAC PF (ß = 0.06; *p* = 0.01) subsequently. None of the novel index subscale scores were significantly associated with WOMAC pain scores. TKA complications, as assessed by TKA-related index subscale,  were not significantly associated with SF-36 or WOMAC domain scores.

**Conclusions:**

Increasing Charlson index as well as novel medical and local musculoskeletal index subscale scores (from novel Arthroplasty  Comorbidity Severity Index) post-TKA correlated with subsequent worsening of physical function and pain outcomes post-TKA. Further studies should examine which comorbidity management could have the greatest impact on these outcomes.

**Electronic supplementary material:**

The online version of this article (doi:10.1186/s12891-016-1261-y) contains supplementary material, which is available to authorized users.

## Background

Over 719,000 total knee arthroplasties (TKAs) were performed in 2010 in the United States [1] and the annual incidence of TKA is expected to increase 7-fold from 2005 to 2030 and exceed 3.5 million annually by 2030 [2]. TKA is associated with impressive gains in the quality of life (QOL) of patients [3]. The benefit achieved with TKA, however, is not universal, nor is it always sustained [4]. Understanding the factors that influence arthroplasty outcomes will allow for better patient selection, provide patients with more realistic expectations, and optimize the procedure’s benefits.

Comorbid conditions may contribute to variation in post-arthroplasty outcome. This is particularly relevant due to the increasing obesity and longevity of the population, which is likely to lead to higher comorbidity in joint arthroplasty recipients. Outcomes, which have thus far been studied in relation to comorbidity, include the length of hospital stay, post-operative complications, short-term readmission and mortality, and short-term pain and physical function. Although it is the perception of both referring physicians and surgeons that higher comorbidity burden relates to worse outcome [5], the literature on this subject is limited and the study results are contradictory.

Higher comorbidity was associated with worse outcome in some studies [6–12] but not in others [13–17]. A recent review of the effect of comorbidity on arthroplasty outcomes concluded “…the overall impact appears to be small” [18]. However, only two of the above-mentioned studies that we found [13, 15] were included in this recent review. The negative and positive studies differed in the confounders adjusted for and the outcome measures, which might explain some of the differences noted between studies.

In addition, most TKA studies have examined outcomes in relation to baseline preoperative comorbidity rather than the change in comorbidity with time postoperatively. A recent paper by Gandhi et al. found that baseline comorbidity, as measured by the Cumulative Illness Rating Scale (CIRS) index, negatively impacted SF-36 Physical Function and Role Physical scores at a mean of 3 years post-arthroplasty [7]. We were interested in assessing whether overall comorbidity, changing through time in the postoperative period, is associated with a decline in function and worse pain outcomes after TKA. In this study, we examined the relationship of the post-operative change in comorbidity with the change in SF-36 and WOMAC physical function and pain scores in the subsequent post-TKA periods. To our knowledge, none of the previous studies have addressed this question.

## Methods

### Study population

We performed a retrospective analysis of prospectively collected data, by additionally performing a detailed chart review of post-TKA patients at the Minneapolis Veterans Administration (VA) Medical Center. The Institutional Review Board (IRB) at the Minneapolis VA approved the study. We included patients from three studies in primary TKA populations at our medical center, since all three studies collected prospective intermediate to long-term pain and function outcome data using Western Ontario and McMaster Universities Osteoarthritis Index (WOMAC) or Short Form-36 (SF36) or both, i.e., outcomes of interest for our study. The patients included in this study were each enrolled in either a randomized study comparing mobile-bearing and fixed-bearing cruciate-substituting TKA (SF-36 and WOMAC) [19], a randomized study comparing all-polyethylene versus metal-backed components in TKA (SF-36) [20], or a cohort study evaluating periprosthetic bone density surrounding tantalum tibial implants in TKA (SF-36 and WOMAC) [21]. Inclusion and exclusion criteria for each study that provided data are detailed in Additional file 1. The patients in each study had completed either both SF-36 and WOMAC surveys, or WOMAC surveys alone, at regular intervals before and approximately every year after their surgery, and had intermediate to long-term follow-up lasting an average of more than 4 years. We included these patients, since patients had completed validated outcomes (main study outcomes), and had a reasonable follow-up.

### Data extraction methods

Using a standardized data extraction form, one trained physician (MH) blinded to the patients’ outcome scores (SF-36 and WOMAC) extracted data from both paper and electronic medical records at the Minneapolis Veterans Affairs (VA) Medical Center. All outpatient and inpatient notes were reviewed from each chart’s inception. All patients obtained their primary care at this medical center or, rarely, at other electronically accessible VA hospitals/clinics. No patients were excluded due to non-availability of records.

Data were organized into sequential periods defined by the interval between surveys, by providing dates for these periods to the abstractor (MH). Clinical data were abstracted starting with the baseline post-operative survey, which was completed 6–12 months after index primary TKA. Sequential periods were of usually 12–20 month duration. Patient demographics (age and gender), date of primary TKA and comorbidities were abstracted. After the completion of data abstraction, these data were merged with previously collected WOMAC and SF-36 scores at patient level and by each period of interest, for statistical analyses.

### Predictors and their definitions

Comorbidity was measured using four measures (Charlson index and three subscale indices of a novel scale) and data were collected on comorbidities for each interval to allow calculation of score for all four comorbidity measures for each interval.

The Charlson index is a weighted scale containing 17 comorbidities expressed as a sum, which has been validated in in- and out-patient settings and widely used for comorbidity adjustment in the medical literature (see Additional file 2) [22, 23]. We developed an alternate novel Minnesota Arthroplasty Comorbidity Index with 3 subscale  indices to understand the impact of comorbidity on function and pain outcomes for two reasons: (1) Charlson index was initially designed to predict mortality and has comorbidities more applicable to inpatients than outpatients and most post-TKA patients are outpatients; and (2) local musculoskeletal comorbidity is likely to impact pain and function after TKA differently than a medical comorbidity and this is not captured in the Charlson index.

We calculated a novel medical/surgical comorbidity severity score, the Minnesota Arthroplasty Comorbidity severity Index (MACI), which measured a greater variety of comorbidities and a more granular categorization of disease severity than the Charlson index (Additional file 3). Comorbidities were divided into three subscales: “Medical index” that included conditions such as diabetes, heart disease, cancer etc., conditions common in patients undergoing TKA(range, 0-47); “Local musculoskeletal index”, which comprised any lower extremity, hip, or spine morbidity (range, 0-10); and “TKA-related index”, which comprised of any adverse event related to their index TKA, including loosening, infection, revision etc. (range, 0-2) (Additional file 3). For each of these measures of comorbidity, we designed a severity scoring system; mild, moderate, severe and very severe categories were scored as 1, 1.5, 2 and 2.5, respectively. This scoring system was based either on specialty- or society-approved guidelines, such as the Kidney Disease Outcomes Quality Initiative (KDOQI) definitions of chronic kidney disease [24], or in their absence, clinical judgment of expert consultants in each specialty (cardiologist, endocrinologist, nephrologist, rheumatologist, orthopedic surgeon, gastroenterologist etc.). The three-component/subscale scores of our novel medical/surgical comorbidity severity index scale, “medical” (range, 0-47);, “local musculoskeletal” (range, 0-10); and “TKA-related”, (range, 0-2); were the predictors of interest. In the case of missing data, values were carried forward from the previous period/interval.

### Outcomes of interest

The SF-36 and WOMAC scores were evaluated from a 6–12 month post-TKA baseline for the first post-TKA time-point and later time-points for subsequent visits. This baseline was chosen since most of the benefit in pain and function in patients undergoing TKA is achieved by 6–12 months post-operatively [7, 25]. Our study objective was to examine the effect of changing comorbidity post-operatively on subsequent pain and function  scores post-TKA, and therefore, we included post-TKA scores as the baseline scores for our study.

The WOMAC is a widely-used self-administered pain and functional ability  scale for patients with lower-extremity osteoarthritis (OA) [26], validated in TKA patients [27]. It assesses pain, physical function, and stiffness, and asks patients about pain or difficulty doing various daily activities, rated on a five-point scale from “none” to “extreme”. Scores for each subscale and total scores were calculated as 0–100 scale; higher scores indicate worse pain, function, and stiffness. We used WOMAC pain and PF subscales as outcomes. We did not use WOMAC stiffness subscale as it has low responsiveness [28].

The SF-36 is a generic health status measure that is scored from 0 to100, with 100 being the best score; the WOMAC is also scored from 0 to100, but 0 is the best score. The eight subscales are: Bodily pain (BP), Physical Functioning (PF), Role Physical (RP), Role Emotional (RE), Social Functioning (SF), Mental Health (MH), Vitality (VT), General Health (GH). We assessed SF-36 PF and SF-BP, since these subscales capture physical functioning and bodily pain, likely impacted by worsening comorbidity post-TKA.

### Statistical analyses

We examined SF-36 PF, SF-36 BP, WOMAC PF and WOMAC pain subscale scores as continuous outcome variables. Model diagnostics including Q-Q plots for residuals and Q-Q plots for random effects were tested. Based on the inherent skewness evident on these plots, we used gamma distribution for response with log link for these continuous variables. We used random intercept gamma generalized linear mixed model with log link to examine the association of increasing comorbidity score (Charlson and three indices from our novel comorbidity measure) with worsening QOL, as measured by the WOMAC and SF-36 PF and pain subscale scores, in the subsequent intervals (see above) [29], that controlled for repeated observations. These reported effects were adjusted for age, baseline respective QOL score and the length of time from index TKA. We present beta coefficients (ß) and *p*-values for these associations. Sensitivity analyses were performed for these associations excluding the single female subject.

## Results

### Clinical characteristics of the study population

We reviewed the charts of 124 patients (123 male, one female) who underwent primary TKA between July 1992 and October 2005 and were followed for a mean of 4.9 years post-operatively (range 1.3–11.4 years; SD 2.8) (Table 1). The average look-back period for preoperative chart review for the assessment of preoperative comorbidity score was 10.5 years (range 2.8–33.8, SD 3.26 years); this score contributed to the immediate post-operative score.

**Table 1:** Patient characteristics

	Overall study Cohort (*n* = 124) Mean ± SD (range)	Cohort with WOMAC and SF-36 data (*n* = 93) Mean ± SD (range)	Cohort with SF-36 data only (*n* = 31) Mean ± SD (range)
Age at index surgery, years	71.7 ± 6.9 (58.6–89.2)	72.6 ± 7.4 (59.8–89.2)	69.0 ± 4.3 (58.6–77.6)
Male/total	123/124	92/93	31/31
Dates index TKA performed	August 1993- October 2005	April 2001- October 2005	August 1993- August 1996
Duration of post-op follow-up, years	4.9 ± 2.9 (1.3–11.4)	3.5 ± 1.2 (1.5–6.0)	9.2 ± 1.9 (1.3–11.4)
Charlson score at post-TKA baseline	0.58 ± 1.00 (0–3)	0.60 ± 0.89 (0–2)	0.57 ± 1.13 (0–3)
Charlson score at interval 5 post-TKA	1.85 ± 1.54 (0–5)	1.53 ± 1.50 (0–5)	2.7 ± 1.38 (1–5)
Medical subscale score of the novel index^a^at post-TKA baseline (range 0-47)	3.7 ± 1.4 (2–6.5)	3.7 ± 1.03 (2.5–5)	3.8 ± 1.6 (2–6.5)
Medical subscale score of the novel index^a^at interval 5 post-TKA (range 0-47)	7.6 ± 3.6 (2–16.5)	7.1 ± 3.5 (2–13.5)	9.0 ± 3.8 (6–16.5)
Local subscale score of the novel index^a^at post-TKA baseline (range 0-10)	5.2 ± 1.2 (2.5–7)	5.1 ± 0.6 (4.5–6)	5.3 ± 1.5 (2.5–7)
Local subscale score of the novel index^a^at interval 5 post-TKA (range 0-10)	1.6 ± 1.2 (0–3)	1.70 ± 1.3 (0–3)	1.4 ± 1.1 (0–3)

^a^ Novel index, novel medical/surgical comorbidity severity index

All patients had completed SF-36 surveys, and 93 of them had completed WOMAC surveys as well (one included study had only SF-36 data). The average age at time of index surgery was 71.7 years (range 58.6–89.2, SD 6.9). For 66 patients, this surgery was the only TKA they had undergone, while the remaining 58 patients underwent opposite side TKA either prior to or after the index TKA surgery. This subsequent surgery was taken into account in our analysis of “Local musculoskeletal” comorbidities, in which arthroplasties on the opposite side were noted. We organized data into intervals defined by the time between sequential WOMAC and SF-36 surveys in a given patient. The average interval between these was 1.4 years.

### Comorbidity during post-operative follow-up

**Fig. 1:**
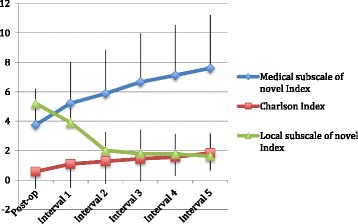
Post-operative interval was 6–12 months post-index TKA and subsequent intervals were 12–24 months long. The intervals were determined based on the time of QOL (SF-36 and WOMAC) assessment, so that comorbidity scores in the study period prior could be correlated with the QOL scores

The average baseline post-operative Charlson score was 0.58 (range 0–3, SD 1.0) (Fig. 1). The average final Charlson score at study follow-up in interval 5 was 1.85 (range 0–5, SD 1.62; Fig. 1). Of the 124 patients, 78 had no change in their Charlson score, and 33 of these maintained a Charlson score of zero for the entire period of study. The most common diagnoses were myocardial infarction (45 patients), diabetes mellitus (41 patients with 27 without and 14 with end-organ damage), congestive heart failure (28 patients), cerebrovascular disease (20 patients with 17 without and 3 with subsequent hemiplegia), and chronic pulmonary disease (18 patients). The mean length of each period ranged from 1 to 1.7 years.

The average baseline and post-operative interval 5 novel comorbidity severity index “medical” subscale scores were 3.7 (range 2–6.5, SD 1.3) and 7.6 (range 2–16.5, SD 3.6), respectively (Table 1). The mean Charlson and novel medical comorbidity severity subscale index scores (medical and local subscales) for each interval are shown (Fig. 1). Patients had a variable follow-up period and some completed their last follow-up visit at interval 2, while others at interval 5. The average “local musculoskeletal” comorbidity subscale scores were 5.2 at baseline post-operative (range 2.5–7, SD 1.2) and 1.6 (range 0–3, SD 1.2) at interval 5 follow-up. The improvement in local musculoskeletal index subscale scores was largely attributable to a proportion of patients undergoing opposite side TKA, which reduced  their high opposite-knee arthritis scores. Only 16 patients scored points in the TKA-related Index, for complications ranging from implant loosening to infection. Seven patients underwent revision, for a cumulative revision rate of 5.6 % in this sample.

### Pain and Function score worsening during TKA follow-up

**Fig. 2:**
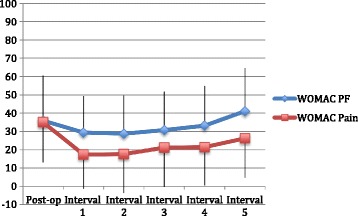
X-axis shows the time since TKA, including the baseline post-operative score at 6–12 months post-index TKA and the subsequent 12–24 month intervals. The Y-axis shows WOMAC pain and physical function (PF) scores, which are out of 100 points, with 0 being the best score and 100 being the worst score

Average baseline post-operative WOMAC pain score was 35 (range 0–100, SD 22.1), which improved to 17.3, in the first post-operative interval and then gradually worsened to follow-up score of 26.2 in post-operative interval 5 (Fig. 2). Mean baseline post-operative WOMAC PF subscale score was 35.9 (range 0–96, SD 24.7), which improved to 29.4 in post-operative interval 1 and then gradually worsened to a follow-up score of 41 in post-operative interval 5.

**Fig. 3:**
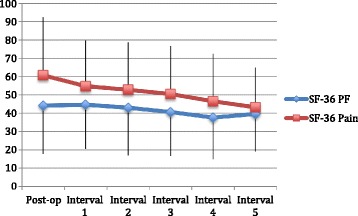
X-axis shows the time since TKA, including the baseline post-operative score at 6–12 months post-index TKA and the subsequent 12–24 month intervals. The Y-axis shows SF-36 physical function (PF) and bodily pain (BP) subscale scores, which are out of 100 points, with 0 being the worst score and 100 being the best score

Mean SF-36 BP score at post-operative baseline was 44.2 (range 0–90, SD 31.7), did not change in post-operative interval 1 to 44.7, and then worsened slightly to 39.6 at the end of follow-up in post-operative interval 5 (range 0–75, SD 21.7) (Fig. 3). Mean SF-36 PF score at post-operative baseline was 60.7 (range 22–100, SD 26.4), worsened to 54.7 in post-operative interval 1 and to 43.2 at the end of follow-up in post-operative interval 5 (range 12–100, SD 20.6).

**Fig. 4:**
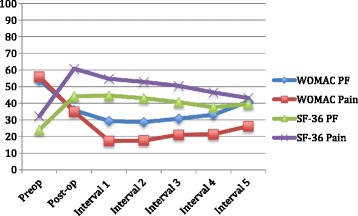
X-axis shows both pre-operative and the post-operative periods since TKA, including the baseline post-operative score at 6–12 months post-index TKA and the subsequent 12–24 month intervals. The Y-axis shows WOMAC pain and physical function (PF) subscale scores and SF-36 PF and bodily pain (BP) subscale scores, which are out of 100 points. On WOMAC, 0 is the best score and 100, the worst score; on SF-36, 0 is the worst and 100, the best score

Figure 4 shows that even though some worsening in WOMAC and SF-36 scores occurred during the postoperative follow-up, scores were still meaningfully better at the interval 5 post-operative, compared to before TKA, primarily due to a significant initial improvement from preoperative to immediate post-operative scores.

### Association of increasing comorbidity post-TKA with subsequent QOL worsening

An increasing Charlson index score was significantly associated with worsening SF-36 PF (ß = -0.07; *p* < 0.0001), SF-36 BP (ß = -0.06; *p* = 0.002), and WOMAC PF (ß = 0.08; *p* < 0.001) scores (Table 2). We found that increasing novel Medical Index score was significantly associated with worsening SF-36 PF scores (ß = -0.03; *p* = 0.002), SF-36 BP (ß = -0.04; *p* < 0.001) and a non-significant trend for worse WOMAC PF scores (ß = 0.02; *p* = 0.11) (Table 2). Worsening of the local musculoskeletal index score (a measure of lower extremity and spine comorbidity) correlated significantly with worsening SF-36 PF (ß = -0.05; *p* = 0.001), SF-36 BP (ß = -0.04; *p* = 0.03) and WOMAC PF (ß = 0.06; *p* = 0.01) (Table 2). The TKA-related index was not associated with worsening any of the WOMAC or SF36 domain scores. Sensitivity analysis that excluded the single female subject led to no change in interpretation of any of these analyses.

**Table 2:** Beta-coefficients (ß) and *p*-values for association between post-operative comorbidity measures and the pain and function measures post-TKA

	Coefficient (95 % CI); *p*-value			
	Charlson Index	Medical subscale Index*	Local Musculoskeletal subscale Index*	TKA-Related subscale Index*
SF36 Physical Functioning (PF)	**-0.07 (-0.11, -0.03);** ***p*** **= 0.001**	**-0.03 (-0.05, -0.01);** ***p*** **= 0.002**	**-0.05 (-0.08, -0.02);** ***p*** **= 0.001**	0.04 (-0.22, 0.13); *p* = 0.64
WOMAC Physical Function (PF)	**0.08 (0.03, 0.12);** ***p*** **< 0.001**	0.02 (-0.004, 0.04); *p* = 0.11	**0.06 (0.01, 0.10);** ***p*** **= 0.01**	0.04 (-0.23, 0.31); *p* = 0.76
SF-36 Bodily Pain (BP)	**-0.06 (-0.09, -0.02);** ***p*** **= 0.002**	**-0.04 (-0.06, -0.02);** ***p*** **< 0.001**	**-0.04 (-0.08, -0.004);** ***p*** **= 0.03 (-**	Not calculable
WOMAC Pain	-0.013 (-0.07, 0.04); *p* = 0.64	-0.01 (-0.04, 0.02); *p* = 0.44	0.02 (-0.04, 0.07); *p* = 0.49	-0.21* (-0.49, 0.08); *p* = 0.15

--, not assessed

SF-36, Short Form 36; WOMAC, Western Ontario McMaster Universities Osteoarthritis index

Significant *p*-values are in bold

*estimate not reliable, since model fit wasn’t good and the model did not converge

*index, novel medical/surgical comorbidity severity index

## Discussion

TKA is the most common arthroplasty procedure worldwide and its utilization is increasing rapidly [1, 2]. Therefore, it is important to understand the variability in TKA outcomes. We developed and tested a novel medical/surgical comorbidity index, the Minnesota Arthroplasty Comorbidity Index (MACI). MACI took into account a greater number of comorbidities as compared to the Charlson index, weighted severity of conditions not only presence (medical index subscale) and included local musculoskeletal index and TKA-related index  subscales. We examined the longitudinal association of medical comorbidity, local musculoskeletal comorbidity and index TKA-related morbidity with worsening of pain and function post-TKA.

As expected and has been shown previously, pain and function scores improved dramatically from pre-operative to the post-operative interval. Most, but not all these gains persisted during intermediate-long post-TKA mean follow-up of 4.9 years post-operatively (range, 1.3–11.4 years). We found that medical and local musculoskeletal comorbidity in the post-TKA period were associated with gradual worsening of function and pain outcomes post-TKA, after a striking initial improvement with TKA, as demonstrated previously with observational studies. Several findings merit further discussion.

A few previous studies have reported that pre-operative comorbidity predicts short-term or very short-term post-arthroplasty outcomes. SooHoo et al. found that the preoperative Charlson score predicted several short-term measures including 90-day readmission postoperatively [12]. Weaver et al. found that in 11,710 veterans with TKA, higher preoperative Deyo-Charlson score (modified version) correlated with longer length of stay and complications 30-days postoperatively [30]. In a study of relatively healthy Australian patients undergoing total hip or knee arthroplasty, baseline individual comorbidity was predictive of change in SF36 physical domain score from the pre- to post-operative period 12-months post-arthroplasty [31]. A previous study showed that SF36 and WOMAC scores declined gradually over the next several years in a study of 551 TKA patients, after an initial post-operative improvement [7]. This decline correlated with pre-operative baseline comorbidity [32]. What we don’t know, however, is whether an increase in comorbidity through time correlates with the decline in SF36 and WOMAC scores post-TKA. Our study advances knowledge  by examining the association of change in comorbidity longitudinally after primary TKA  long-term with pain and function outcomes post-TKA. In our study, we examined change in comorbidity post-operatively over time, and examined that as the predictor of pain and function in subsequent periods of observation. We found that increasing comorbidity post-TKA was significantly associated with future worsening of pain and physical function after primary TKA.

Our findings must be compared to a cross-sectional study done previously on this topic. Dunbar et al. performed a cross-sectional survey 6.7 years post-TKA patients, and used the modified patient-reported Charnley Classification [33]. The Class C patients (unilateral TKA and remote arthritis and/or a medical condition that affected their ability to ambulate) had statistically significantly worse QOL scores demonstrating that a simplified measure of comorbidity correlated with a decline in patients’ QOL. The cross-sectional study could not address this relationship beyond a correlation, since there was only one measurement post-TKA and both comorbidity and QOL were examined at the same time. Our study extends these findings of association of post-TKA comorbidity with post-TKA outcomes, by studying them longitudinally for the first time, controlling for baseline post-operative pain/function and other confounders, assessing the worsening or comorbidity in periods prior to the assessment of pain and function and using multiple measures of comorbidity. These findings are important for several reasons.

The clinical relevance of our study findings is that increasing comorbidity in post-TKA years might explain declining pain and function outcomes in patient subgroups ﻿in the years after TKA. Surgeons and patients should discuss this during the informed consent process, so that expectations are realistic and patients are empowered with this information. Close monitoring and early intervention to treat new/worsening comorbidities might help in reducing the impact of comorbidities on pain and function outcomes in the long-term follow-up post-TKA.

Instead of a more simplified measure as described above, we used two weighted measures of comorbidity in our study, the Charlson index, and our novel arthroplasty comorbidity severity index. The method of measuring and reporting comorbidities in previous studies varies significantly. Many comorbidity indices tabulate the comorbidities as a single score with a sum. Studies commonly stratify patient comorbidity sum as dichotomous variable, e.g. <4 vs. 4 or more comorbidities [34]. The summation and stratification methods, however, do not account for the severity of various illnesses. To address this issue, weighted, validated comorbidity measures have been created, the best known of which is the Charlson index [22, 35]. Charlson index correlates with mortality, hospital readmission, length of stay, post-operative complications, progression-free survival in cancer patients, and disability [35, 36]. It has been validated in various conditions, e.g., pneumonia, heart disease, spine surgery and amputation [36].

In this study, we explored the relationship between comorbidity and postoperative worsening of QOL measures. We examined a more comprehensive spectrum of comorbidities than the Charlson index by creating a novel medical/surgical comorbidity index focusing on common comorbidities of TKA patients, which consisted of three component subscales: medical, local musculoskeletal (lower extremity and spine morbidity), and TKA-related comorbidities (e.g. problems with the TKA itself). Our medical/surgical index “medical” subscale examined 25 common comorbidity categories compared to 13 in the Charlson index, and while there is some overlap, we included several more common diagnoses in the ambulatory patient populations such as those undergoing TKA (e.g. hypertension), rather than more severe (but much less common) diagnoses as in the Charlson index (e.g. AIDS or “second metastatic solid tumor”); the score ranges 0-47. We also included several diagnoses of a more function-limiting nature, such as hearing and vision problems or alcohol abuse. We found that, both the Charlson Index and our “medical” subscale score from the novel index correlated with worsening SF36 PF and WOMAC PF. Future studies should compare their discriminative abilities for TKA outcomes to understand how to best use these measures in arthroplasty patient populations.

Interestingly, our local musculoskeletal index subscale score also correlated significantly with SF36 PF and WOMAC PF. This index included opposite knee, hip, and spine arthritis, as well as local problems such as neuropathy and vascular disease. This suggests that lower extremity and spine issues, though localized, are important determinants of lower extremity as well as global physical function. Not surprisingly, studies have shown that ipsilateral hip arthritis/pain, low back pain, and contralateral knee arthritis/pain might contribute to index knee pain [37–40]. And because the non-overlapping TKA-related Index included all TKA-related problems such as loosening or infection, this finding the association of local musculoskeletal index subscale score with SF36 PF and WOMAC PF cannot be attributed to the arthroplasty itself.

We also found that patients with worsening local musculoskeletal index subscale score  had more pain in the subsequent periods post-operatively, indicated by a significant association with SF-36 pain scores. Pain correlates with worse functional outcome [41]. Post-TKA pain is likely multifactorial, related to more than just the index knee replacement. Increasing comorbidity post-TKA may interfere with optimal rehabilitation leading to suboptimal recovery and residual pain and functional limitation. It remains to be seen whether targeted pain control and rehabilitation protocols, and optimal perioperative management of comorbid conditions can impact post-TKA pain and function outcomes.

Our study has some limitations. We had a relatively small sample size of 124 patients, of which 123 were men. We considered limiting the analyses to only men, but decided to analyze the entire sample, since it makes our findings more representative of the entire VA patient population (95–99 % men and 1–5 % women), not just male veterans. However, we conducted sensitivity analyses limited to men, which had the same conclusions as the total cohort (as expected). We controlled for age, length of time since surgery, and baseline QOL values, but not for other factors. Some patients, for example, had undergone only unilateral TKA, while others had (either before or after the index surgery) undergone second TKAs on the opposite knee. This could have impacted the pain and function scores, particularly in patients who had contralateral arthritis but had not had a contralateral TKA; it would be interesting to explore this relationship. We did, however, attempt to control for this by measuring lower-extremity-related comorbidity as described above. Our patients had been enrolled in three different studies, two randomized and one cohort, which may have introduced sample heterogeneity and we were not able to control for it. The mean follow-up interval prior to each assessment was 12–20 months; while slight differences in time-periods can lead to some heterogeneity in parameter estimates, we used a repeated measures mixed model linear regression and comorbidity assessments were performed for the period prior to each QOL assessment, partially accounting for this.

Comorbidities were abstracted by a physician’s chart review of their documentation in health care provider notes. The accuracy of this approach of comorbidity assessment in a retrospective study can not be assessed due to the absence of a gold standard, such as patient examination in a prospective cohort study. Despite suspected under-documentation of some comorbidities (especially psychological), we suspect that this method of capturing comorbidity may be more accurate than capturing diagnostic codes for comorbidity from administrative databases. Lastly, our newly developed scale is not validated as yet; test of reliability and several other aspects of validity are needed before any further use of the MACI scale. However, additional value over the existing comorbidity scales wasn’t evident in this study cohort. It is possible that this scale may be more relevant for patients undergoing revision arthroplasty or other joint procedures.

Our study has several strengths. First, our population was well suited to this investigation. Medical comorbidities are prevalent in the veteran population, since 72 % of VA patients have at least one chronic condition [37]. This is significantly higher than the 47 % of Americans overall with at least one chronic condition [38]. We performed a complete review of both inpatient and outpatient records. Zhang et al. reported that compared to a look-back period of 1 year of inpatient data, a longer look-back period using multiple sources of data (2 years of inpatient plus 1 year of outpatient) significantly improved the mortality prediction of the Charlson index [39]. We reviewed more data than this in our patients, with an average look-back period of 10.5 years (range 2.8–33.8, SD 3.26 years), examined a long-period for assessing baseline comorbidity. This is a longer follow-up than in many post-arthroplasty studies of comorbidity, which mostly ranged from 30 days to 2 years [9, 30, 34, 40, 42, 43]. In comparison to certain physician-derived scores such as the Knee Society Score, which requires a surgeon’s assessment, [44] we used validated, patient self-reported instruments (the WOMAC and SF36) to measure post-TKA outcomes. In TKA patients, WOMAC may have a bigger effect size and overall responsiveness than generic surveys [45] such as SF-36 and better correlate with knee-specific complaints [46].

## Conclusions

In conclusion, we used validated measures of comorbidity [22, 23], and pain/function/QOL outcomes, the WOMAC [26] and the SF-36 [47], to measure their relationship in a post-TKA patient population. Our study provides further evidence that medical comorbidity load increased with longer observation times after the primary TKA. We found that some (but not all) of the initial gain/improvement of pain and function with primary TKA was lost over time. We showed that worsening medical comorbidity over time after the knee arthroplasty could explain some of the post-operative decline in patient outcomes of pain and function. Local musculoskeletal index (including lower extremity, spine etc.) also influenced pain and function outcomes, in the intermediate-long term follow-up after primary TKA. We developed MACI, a novel medical/surgical severity index for patients with arthroplasty, incorporating medical, local musculoskeletal comorbidity and TKA-related comorbidity subscales, which will need validation and further testing.

## Additional files

Additional file 1: Inclusion and exclusion criteria for included studies. (DOCX 17 kb)
Additional file 2: Charlson index based on the presence of each diagnosis [19]. (DOCX 13 kb)
Additional file 3: Novel Minnesota Arthroplasty Comorbidity Index (MACI) with components for medical index, local index and TKA-related index sub-scores. (DOCX 15 kb)

## Abbreviations

BP: Bodily pain
CI: Confidence interval
MACI: Minnesota Arthroplasty Comorbidity Index
OA: Osteoarthritis
PF: Physical Functioning
QOL: Quality of life
RP: Role Physical
SF36: Short Form-36
TKA: Total knee arthroplasty
VA: Veterans Affairs
WOMAC: Western Ontario and McMaster Universities Osteoarthritis Index

## References

[1] HCUP. Number of all-listed procedures for discharges from short-stay hospitals, by procedure category and age: United States, 2010. http://www.cdc.gov/nchs/data/nhds/4procedures/2010pro4_numberprocedureage.pdf. Accessed 26 Sept 2016.

[2] Kurtz S, Ong K, Lau E, Mowat F, Halpern M (2007). Projections of primary and revision hip and knee arthroplasty in the United States from 2005 to 2030. J Bone Joint Surg Am.

[3] Kane RL, Saleh KJ, Wilt TJ, Bershadsky B (2005). The functional outcomes of total knee arthroplasty. J Bone Joint Surg Am.

[4] Benjamin J, Johnson R, Porter S (2003). Knee scores change with length of follow-up after total knee arthroplasty. J Arthroplasty.

[5] Sturmer T, Dreinhofer K, Grober-Gratz D, Brenner H, Dieppe P, Puhl W, Gunther KP (2005). Differences in the views of orthopaedic surgeons and referring practitioners on the determinants of outcome after total hip replacement. J Bone Joint Surg (Br).

[6] Fisher DA, Dierckman B, Watts MR, Davis K (2007). Looks good but feels bad: factors that contribute to poor results after total knee arthroplasty. J Arthroplasty.

[7] Gandhi R, Dhotar H, Razak F, Tso P, Davey JR, Mahomed NN. Predicting the longer term outcomes of total knee arthroplasty. Knee. 2010;17(1):15-18. 10.1016/j.knee.2009.06.003 19589683

[8] Jain NB, Guller U, Pietrobon R, Bond TK, Higgins LD (2005). Comorbidities increase complication rates in patients having arthroplasty. Clin Orthop Relat Res.

[9] Lingard EA, Katz JN, Wright EA, Sledge CB (2004). Predicting the outcome of total knee arthroplasty. J Bone Joint Surg Am.

[10] Rajgopal V, Bourne RB, Chesworth BM, Macdonald SJ, McCalden RW, Rorabeck CH. The impact of morbid obesity on patient outcomes after total knee arthroplasty. J Arthroplasty. 2008;23(6):795-800. 10.1016/j.arth.2007.08.005 18534516

[11] Singh JA. Effect of comorbidity on quality of life of male veterans with prevalent primary total knee arthroplasty. Clin Rheumatol. 2009;28(9):1083-89. 10.1007/s10067-009-1195-y PMC2950804 19449146

[12] SooHoo NF, Lieberman JR, Ko CY, Zingmond DS (2006). Factors predicting complication rates following total knee replacement. J Bone Joint Surg Am.

[13] Fortin PR, Clarke AE, Joseph L, Liang MH, Tanzer M, Ferland D, Phillips C, Partridge AJ, Belisle P, Fossel AH (1999). Outcomes of total hip and knee replacement: preoperative functional status predicts outcomes at six months after surgery. Arthritis Rheum.

[14] Hawker G, Wright J, Coyte P, Paul J, Dittus R, Croxford R, Katz B, Bombardier C, Heck D, Freund D (1998). Health-related quality of life after knee replacement. J Bone Joint Surg Am.

[15] Jones CA, Voaklander DC, Johnston DW, Suarez-Almazor ME (2001). The effect of age on pain, function, and quality of life after total hip and knee arthroplasty. Arch Intern Med.

[16] Naylor JM, Harmer AR, Heard RC (2008). Severe other joint disease and obesity independently influence recovery after joint replacement surgery: an observational study. Aust J Physiother.

[17] Nunez M, Nunez E, Segur JM, Macule F, Sanchez A, Hernandez MV, Vilalta C (2007). Health-related quality of life and costs in patients with osteoarthritis on waiting list for total knee replacement. Osteoarthritis Cartilage.

[18] Jones CA, Beaupre LA, Johnston DW, Suarez-Almazor ME: Total joint arthroplasties: current concepts of patient outcomes after surgery. Rheum Dis Clin North Am 2007, 33(1):71-86. 10.1016/j.rdc.2006.12.008 17367693

[19] Gioe TJ, Glynn J, Sembrano J, Suthers K, Santos ER, Singh J (2009). Mobile and fixed-bearing (all-polyethylene tibial component) total knee arthroplasty designs. A prospective randomized trial. J Bone Joint Surg Am.

[20] Gioe TJ, Stroemer ES, Santos ER (2007). All-polyethylene and metal-backed tibias have similar outcomes at 10 years: a randomized level I [corrected] evidence study. Clin Orthop Relat Res.

[21] Harrison AK, Gioe TJ, Simonelli C, Tatman PJ, Schoeller MC (2010). Do porous tantalum implants help preserve bone?: evaluation of tibial bone density surrounding tantalum tibial implants in TKA. Clin Orthop Relat Res.

[22] Charlson ME, Pompei P, Ales KL, MacKenzie CR (1987). A new method of classifying prognostic comorbidity in longitudinal studies: development and validation. J Chronic Dis.

[23] Charlson ME, Sax FL, MacKenzie CR, Braham RL, Fields SD, Douglas RG (1987). Morbidity during hospitalization: can we predict it?. J Chronic Dis.

[24] National Kidney Foundation. K/DOQI clinical practice guidelines for chronic kidney disease: evaluation, classification, and stratification. Am J Kidney Dis. 2002; 39(2 Suppl 1):S1-266. 11904577

[25] Davis AM, Agnidis Z, Badley E, Kiss A, Waddell JP, Gross AE (2006). Predictors of functional outcome two years following revision hip arthroplasty. J Bone Joint Surg Am.

[26] Bellamy N, Buchanan WW, Goldsmith CH, Campbell J, Stitt LW (1988). Validation study of WOMAC: a health status instrument for measuring clinically important patient relevant outcomes to antirheumatic drug therapy in patients with osteoarthritis of the hip or knee. J Rheumatol.

[27] Kurtz S, Mowat F, Ong K, Chan N, Lau E, Halpern M (2005). Prevalence of primary and revision total hip and knee arthroplasty in the United States from 1990 through 2002. J Bone Joint Surg Am.

[28] Angst F, Aeschlimann A, Michel BA, Stucki G (2002). Minimal clinically important rehabilitation effects in patients with osteoarthritis of the lower extremities. J Rheumatol.

[29] Lindstrom ML, Bates DM (1988). Newton-Raphson and EM algorithms for linear mixed-effects models for repeated measures data. J Am Stat Assoc.

[30] Weaver F, Hynes D, Hopkinson W, Wixson R, Khuri S, Daley J, Henderson WG (2003). Preoperative risks and outcomes of hip and knee arthroplasty in the Veterans Health Administration. J Arthroplasty.

[31] Harse JD, Holman CD (2005). Charlson’s Index was a poor predictor of quality of life outcomes in a study of patients following joint replacement surgery. J Clin Epidemiol.

[32] Linn BS, Linn MW, Gurel L (1968). Cumulative illness rating scale. J Am Geriatr Soc.

[33] Dunbar MJ, Robertsson O, Ryd L (2004). What’s all that noise? The effect of co-morbidity on health outcome questionnaire results after knee arthroplasty. Acta Orthop Scand.

[34] Wasielewski RC, Weed H, Prezioso C, Nicholson C, Puri RD (1998). Patient comorbidity: relationship to outcomes of total knee arthroplasty. Clin Orthop Relat Res.

[35] Extermann M (2000). Measuring comorbidity in older cancer patients. Eur J Cancer.

[36] de Groot V, Beckerman H, Lankhorst GJ, Bouter LM (2003). How to measure comorbidity. a critical review of available methods. J Clin Epidemiol.

[37] Yu W, Ravelo A, Wagner TH, Phibbs CS, Bhandari A, Chen S, Barnett PG (2003). Prevalence and costs of chronic conditions in the VA health care system. Med Care Res Rev.

[38] Hoffman C, Rice D, Sung HY (1996). Persons with chronic conditions. Their prevalence and costs. JAMA.

[39] Zhang JX, Iwashyna TJ, Christakis NA (1999). The performance of different lookback periods and sources of information for Charlson comorbidity adjustment in Medicare claims. Med Care.

[40] Ayers DC, Franklin PD, Ploutz-Snyder R, Boisvert CB (2005). Total knee replacement outcome and coexisting physical and emotional illness. Clin Orthop Relat Res.

[41] Dahlen L, Zimmerman L, Barron C (2006). Pain perception and its relation to functional status post total knee arthroplasty: a pilot study. Orthop Nurs.

[42] Jones CA, Beaupre LA, Johnston DW, Suarez-Almazor ME (2005). Total joint arthroplasties: current concepts of patient outcomes after surgery. Clin Geriatr Med.

[43] MacWilliam CH, Yood MU, Verner JJ, McCarthy BD, Ward RE (1996). Patient-related risk factors that predict poor outcome after total hip replacement. Health Serv Res.

[44] Brinker MR, Lund PJ, Barrack RL (1997). Demographic biases of scoring instruments for the results of total knee arthroplasty. J Bone Joint Surg Am.

[45] Wright JG, Young NL (1997). A comparison of different indices of responsiveness. J Clin Epidemiol.

[46] Hawker G, Melfi C, Paul J, Green R, Bombardier C (1995). Comparison of a generic (SF-36) and a disease specific (WOMAC) (Western Ontario and McMaster Universities Osteoarthritis Index) instrument in the measurement of outcomes after knee replacement surgery. J Rheumatol.

[47] Ware JE, Kosinski M, Dewey JE (2001). How to Score Version 2 of the SF-36® Health Survey (Standard & Acute Forms).

