# Supplementary material for: Increasing comorbidity is associated with worsening physical function and pain after primary total knee arthroplasty
Source: BMC Musculoskelet Disord. 2016 Oct 7;17:421. doi: 10.1186/s12891-016-1261-y (PMC5055707; doi:10.1186/s12891-016-1261-y)
Supplement: Additional file 1: — Inclusion and exclusion criteria for included studies. (DOCX 17 kb) [file 12891_2016_1261_MOESM1_ESM.docx]

**Additional File 1.** Inclusion and exclusion criteria for included studies

|  | Inclusion criteria | Exclusion criteria |
| --- | --- | --- |
| Mobile and Fixed-Bearing (All-Polyethylene Tibial Component) Total Knee Arthroplasty Designs [1] | - 60 to 85 years of age who met the generally accepted clinical and radiographic criteria for treatment with a primary total knee arthroplasty. | - Substantial angular deformity that clearly required an osteotomy or use of a more constrained design - Those in whom bone loss necessitated structural grafting or modular augmentation - Mental function precluding response to standard questionnaires. |
| All-polyethylene and metal-backed tibias have similar outcomes at 10 years: a randomized level I [corrected] evidence study. Clin Orthop Relat Res. 2007;455:212-8. [2] | - 60 years and older having TKAs not necessitating bone grafting, modular stems or augments, or more constrained designs. |  |
| Do porous tantalum implants help preserve bone?: evaluation of tibial bone density surrounding tantalum tibial implants in TKA. Clin Orthop Relat Res. 2010;468(10):2739-45 [3] | - Younger than 62 years undergoing primary TKA between 2005 and 2007 - Patients with a prior contralateral TKA were not excluded | - Known metabolic bone disease, - Taking medications affecting BMD such as bisphosphonates, calcitonin, and hormone replacement (calcium and vitamin D were not excluded), - Preoperative deformity judged to require modular stems, augmentation, or a degree of constraint not offered in the studied design. |
| 1. Gioe TJ, Glynn J, Sembrano J, Suthers K, Santos ER, Singh J: **Mobile and fixed-bearing (all-polyethylene tibial component) total knee arthroplasty designs. A prospective randomized trial**. *J Bone Joint Surg Am* 2009, **91**(9):2104-2112.  2. Gioe TJ, Stroemer ES, Santos ER: **All-polyethylene and metal-backed tibias have similar outcomes at 10 years: a randomized level I [corrected] evidence study**. *Clin Orthop Relat Res* 2007, **455**:212-218.  3. Harrison AK, Gioe TJ, Simonelli C, Tatman PJ, Schoeller MC: **Do porous tantalum implants help preserve bone?: evaluation of tibial bone density surrounding tantalum tibial implants in TKA**. *Clin Orthop Relat Res* 2010, **468**(10):2739-2745. | | |
