# Supplementary material for: Increasing comorbidity is associated with worsening physical function and pain after primary total knee arthroplasty
Source: BMC Musculoskelet Disord. 2016 Oct 7;17:421. doi: 10.1186/s12891-016-1261-y (PMC5055707; doi:10.1186/s12891-016-1261-y)
Supplement: Additional file 2: — Charlson index based on the presence of each diagnosis [19]. (DOCX 13 kb) [file 12891_2016_1261_MOESM2_ESM.docx]

**Additional File 2.** Charlson index based on the presence of each diagnosis [19]

| 1 point | 2 points | 3 points | 4 points |
| --- | --- | --- | --- |
| - Myocardial infarct - Congestive heart failure - Peripheral vascular disease - Cerebrovascular disease - Dementia - Chronic pulmonary disease - Connective tissue disease - Ulcerative disease - Mild liver disease - Diabetes (without complications) | - Diabetes with end organ damage - Hemiplegia - Moderate or severe renal disease - 2^nd^ Solid tumor (non metastatic) - Leukemia - Lymphoma or Multiple Myeloma | - Moderate or severe liver disease | - 2^nd^ Metastatic solid tumor - AIDS |
