# Supplementary material for: Increasing comorbidity is associated with worsening physical function and pain after primary total knee arthroplasty
Source: BMC Musculoskelet Disord. 2016 Oct 7;17:421. doi: 10.1186/s12891-016-1261-y (PMC5055707; doi:10.1186/s12891-016-1261-y)
Supplement: Additional file 3: — Novel Minnesota Arthroplasty Comorbidity Index (MACI) with components for medical index, local index and TKA-related index sub-scores. (DOCX 15 kb) [file 12891_2016_1261_MOESM3_ESM.docx]

**Additional File 3.** Novel Minnesota Arthroplasty Comorbidity Index (MACI) with components for medical index, local index and TKA-related index sub-scores

|  | Diagnosis | Severity | | | |
| --- | --- | --- | --- | --- | --- |
|  |  | Mild (1 point) | Moderate (1.5 points) | Severe (2 points) | Very Severe (2.5 points) |
| Medical Index | Obesity | BMI 25-29.9 | BMI 30-34.9 | BMI 35-39.9 | BMI >40 |
|  | Congestive Heart Failure | EF >50% | EF = 30-50% | EF <30% |  |
|  | Coronary Artery Disease | Positive stress test | MI, PTCA, stent; add'l intervention adds 0.5 pts | Coronary artery bypass graft |  |
|  | Heart valve abnormality | >1.5 cm/s^2^ | 1-1.5 cm/s^2^ | <1 cm/s^2^ or surgery |  |
|  | Arrhythmia | Bradycardia | Atrial fibrillation /flutter | Ventricular tachycardia, pacemaker, ICD, ablation |  |
|  | COPD | FEV1/FVC < 70% or FEV1 ≥ 80% of predicted | FEV1/FVC <70% or FEV1 =50-80% of predicted | FEV1/FVC <70% or FEV1 =30-50% of predicted | FEV1 <30% of predicted |
|  | Interstitial Lung Disease | No home O2 or hospitalization | Home O2 or hospitalization | Home O2 plus intubation or MICU admission |  |
|  | Pneumonia | Not hospitalized | Hospitalized | Intubated or MICU |  |
|  | Asthma | Not hospitalized | Hospitalized | Intubated or MICU |  |
|  | Liver disease | MELD <9 | MELD =10-19 | MELD =20-30 | MELD >30 |
|  | Gastrointestinal bleed | Not hospitalized | Hospitalized or transfused | Surgical intervention |  |
|  | Chronic kidney disease | GFR =60-89 ml/min | GFR =30-59 ml/min | GFR =15-29 ml/min | <15 ml/min or hemodialysis |
|  | Hypertension | Consistently < 135/85 mm Hg | Uncontrolled |  |  |
|  | Symptomatic DVT or PE | DVT |  | PE |  |
|  | Arterial vascular disease | Abnormal imaging |  | Surgical intervention |  |
|  | CVA/TIA | TIA | CVA without sequelae | CVA with sequelae |  |
|  | Diabetes Mellitus | Oral or diet control, none of the triopathy* | Insulin or one of the triopathy* | Triopathy* (nephropathy, retinopathy, neuropathy) |  |
|  | Anemia | Hemoglobin 10- 12 gm/dl | Hemoglobin 8- 10 gm/dl | Hemoglobin 6.5- 8 gm/dl | Hemoglobin <8 gm/dl |
|  | Malignancy | Skin, no metastases |  | Chemo/radiation/surgery | Metastatic or palliative |
|  | Neuropsychiatric | Depression, PTSD, anxiety | Mental Health referral | Hospitalization |  |
|  | Inflammatory arthritis | Gout, Pseudogout |  | RA, Spondyloarthropathy |  |
|  | Alcohol abuse | Present |  |  |  |
|  | Falls | Present |  |  |  |
|  | Vision problems | Present |  |  |  |
|  | Hearing problems | Present |  |  |  |
| Local  Index | Knee arthritis | X-ray or chart documentation | X-ray plus chart documentation | Surgery |  |
|  | Hip arthritis | X-ray or chart documentation | X-ray plus chart documentation | Surgery |  |
|  | Spine arthritis | X-ray or chart documentation | X-ray plus chart documentation | Surgery |  |
|  | Peripheral vascular disease | Abnormal ABI |  | Surgery |  |
|  | Neuropathy | Present |  |  |  |
|  | Any surgery | Present |  |  |  |
| TKA-related  Index | TKA issue | Loosening, lucency, myositis ossificans, manipulation under anesthesia, patella fracture, intraoperative femur fracture, infection treated conservatively | Poly exchange, debridement | Explant, revision |  |

BMI: body mass index. EF: ejection fraction. MI: myocardial infarction. PTCA: percutaneous transluminal coronary angioplasty. ICD: implantable cardioverter defibrillator. COPD: chronic obstructive pulmonary disease. FEV1: forced expiratory volume in 1 minute. FVC: forced vital capacity. O2: oxygen. MICU: medical intensive care unit. MELD: model for end-stage liver disease. GFR: glomerular filtration rate. DVT: deep venous thrombosis. PE: pulmonary embolus. CVA: cerebrovascular accident. TIA: transient ischemic attack. PTSD: post-traumatic stress disorder. RA: rheumatoid arthritis. ABI: ankle-brachial index.

*Triopathy, defined as the presence of nephropathy, retinopathy and neuropathy
